# An Evaluation of High-Risk HPV in Squamous Cell Carcinomas of the Lip in a South African Cohort

**DOI:** 10.1007/s12105-024-01639-0

**Published:** 2024-05-06

**Authors:** Sharon N. Harbor, Johann W. Schneider, Nadine Solomons, Micheline Sanderson, Amir H. Afrogheh

**Affiliations:** 1 Division of Anatomical Pathology, Stellenbosch University, Cape Town, South Africa; 2 Department of Oral and Maxillofacial Pathology, National Health Laboratory Service, University of the Western Cape, Cape Town, South Africa; 3 National Health Laboratory Service, Tygerberg Hospital, Cape Town, South Africa

**Keywords:** Lip, Squamous cell carcinoma, Human papilloma virus, p16, HPV DNA PCR, mRNA

## Abstract

**Background:**

To determine the prevalence of HR-HPV in a series of lip SCC from South African patients, using currently accepted HPV-testing methodologies and to define the clinical and histomorphologic features of HPV-associated lip SCC.

**Methods:**

Fifty SCC of lip and 50 control cases were tested for HR-HPV using p16 and HR-HPV DNA PCR. p16-equivocal/positive and HPV DNA PCR-positive SCC were further evaluated for the expression of HPV-16 and HPV-18 mRNA transcripts using reverse transcription quantitative real-time polymerase chain reaction (RT-qPCR) to confirm transcriptionally active HPV.

**Results:**

p16 was positive in 22% (*n* = 11) and equivocal in 4% (*n* = 2) of the SCC. One p16-positive case showed positivity for both HPV-16 DNA and HPV-16 E6/E7 mRNA transcripts (HPV prevalence rate of 2%). The HPV-positive case was non-keratinizing and occurred in an 80-year-old female. The two p16-equivocal cases were HR-HPV DNA positive and mRNA PCR negative. p16 was found to have a positive predictive value of 9%.

**Conclusion:**

Findings from our cohort of lip SCC suggest that HR-HPV may have an insignificant role in the pathogenesis of SCC at this site. Due to its low ppv, p16 is insufficient to establish HR-HPV infection in SCC of the lip. The combination of p16 and DNA PCR appears to correlate with the presence of transcriptionally active virus. HPV E6/E7 mRNA detection is the gold standard for identifying HR-HPV. mRNA testing is not widely available in sub-Saharan Africa due to technical and financial constraints; however, the test appears to be of great value in p16-equivocal lip SCC.

## Introduction

In 2020, 747,316 newly reported cases of lip, oral cavity, and pharyngeal cancer were estimated globally. During the same time period, there were 367,285 reported deaths with an age-standardized mortality rate (ASR) of 3.9 cases per 100,000 population [1]. Notably lip and oral cavity cancer exhibited the highest mortality rate (1.9), followed by nasopharyngeal (0.88), oropharyngeal (0.51), hypopharyngeal (0.23), and salivary gland cancer (0.23) [1]. In 2020, the incidence of oral and lip cancers in Africa was 14,286 [1]. Lip cancers are frequently grouped with oral cancers and are believed to constitute 25% of all oral cancers [2]. The true prevalence of lip cancer in Africa is unknown, and accurate data are lacking.

Squamous cell carcinoma (SCC) is the most common malignancy of the lip and is usually seen on the skin, vermillion border, or mucosal surface of the lower lip [3]. Risk factors for mucosal SCC include tobacco smoking and alcohol, while fair skin and long-term exposure to ultraviolet light are risk factors for vermillion border SCC [3, 4].

The identification of high-risk human papilloma virus (HR-HPV) as a risk factor in some human malignancies has resulted in a better understanding of the behavior and treatment of HPV-associated cancers [3, 5]. The integration of HR-HPV DNA types such as 16 and 18 into the host genome and the consequent sustained expression of the E6 and E7 oncoproteins is characteristic of HPV-associated malignancies. The E6 and E7 oncoproteins induce the degradation of the tumor suppressor proteins, p53 and pRb, resulting in cellular proliferation and accumulation of abnormal cells. E6 degrades p53, which leads to inhibition of apoptosis and subsequent cellular longevity and immortalization. Conversely, E7 causes functional inactivation of pRb, which prevents pRb from binding to the E2F family of transcription factors. The latter event results in overexpression of p16 tumor suppressor protein in the nucleus and cytoplasm of virally infected cells, detectable by immunohistochemistry [6]. Thus, p16 overexpression is generally correlated with the presence of HR-HPV and is used as a surrogate marker for HR-HPV infection [6, 7].

HR-HPV is a well-established causative agent in oropharyngeal squamous cell carcinoma (OPSCC) [8, 9]. The presence of transcriptionally active HR-HPV in OPSCC is generally associated with better overall survival than HPV-negative tumors. As a result, traditional paradigms relating to the management of OPSCC have changed [10, 11]. HR-HPV status has been investigated as a prognostic indicator in non-oropharyngeal head and neck squamous cell carcinomas (HNSCC) [12]. HR-HPV has been detected in other head and neck sites including the sinonasal cavity where up to 25.5% of carcinomas are reportedly HPV-related [13]. A large meta-analysis found the prevalence of HPV in the oral cavity to be 24.2% and larynx 22.1% [14].

HPV has been postulated as a possible etiologic factor in SCC of the lip. Studies investing the role of HR-HPV in lip SCC have yielded conflicting results with detection rates ranging from 0 to 100% (Table 1) [12, 15–18]. The variations in detection rates across these studies may be attributed to disparities in sample sizes and methodologies employed for HPV detection. Consequently, the true prevalence of HR-HPV and its role in the etiopathogenesis of SCC of the lip remains unclear and warrants further investigation. This study aims to establish the presence or absence of HR-HPV in SCC of the lip in a South African cohort using currently accepted HPV-testing methodologies, including mRNA PCR.

**Table 1:** Reported prevalence of HR-HPV in SCC of the lip

Author	Country	Year	Lip SCC	HPV Positive	Method	HPV-16	HPV-18	Overall %
Harbor et al. (Current Study)	South Africa	2023	50	1	p16, PCR, mRNA	1	0	2%
Mohamed et al. [15]	Sudan	2021	8	0	PCR	0	0	0
Tealab et al. [16]	Egypt	2019	29	14	p16	NK	NK	48%
Wookey et al. [12]	USA	2019	467	35	ND^d^	NK	NK	7.5%
Ndiaye et al.^a^ [18]	Multiple^c^	2014	5478^b^	NK	PCR^e^	NK	NK	19.2%
Mitsuishi et al. [17]	Japan	2005	5	5	PCR	0	3	100%

*PCR* polymerase chain reaction, *NK* not known, ND not described

^a^Systematic review

^b^The sample contains a combination of SCC from the lip and other parts of the oral cavity

^c^Participants were selected from numerous countries

^d^Study is based on reported SEER data for which HPV detection methods are not described

^e^The review also evaluated p16 and E6/E7 mRNA but primarily in oropharyngeal squamous cell carcinomas

## Materials and Methods

### Study Setting

The study took place in the Division of Anatomical Pathology at the National Health Laboratory Service (NHLS), Tygerberg Hospital (TBH), Cape Town, South Africa. TBH is a public tertiary hospital and the second largest hospital in South Africa.

### Study Design

This retrospective, observational case series was conducted under the auspices of the Health Research Ethics Committee (HREC) of the University of Stellenbosch (ref #: S20/06/133), in compliance with the South African National Health Act No. 61 of 2003 and in adherence to the tenets of the Declaration of Helsinki. A search of the National Health Laboratory Service (NHLS) pathology information system was performed. Sequential surgical samples of 50 SCC of the lip and 50 demographically unmatched controls of non-neoplastic lesions of the lip diagnosed over an 11-year period (2009–2019) at TBH were selected. The formalin-fixed, paraffin-embedded (FFPE) tissue blocks, and the corresponding hematoxylin and eosin-stained tissue sections of the SCC and control group were retrieved from the archive of the Division of Anatomical Pathology, NHLS, TBH. Hematoxylin and eosin-stained sections were reviewed to confirm the histopathologic diagnosis, assess the histomorphologic features of each tumor, and determine the biopsy site on the lip (vermillion border, skin, or mucosa). The tumors were graded as Grade 1 (well differentiated), Grade 2 (moderately differentiated), or Grade 3 (poorly differentiated) [19]. To enhance reproducibility and standardize our grading system within the context of this study, we defined Grade 1 as cases characterized by more than 50% keratinization, Grade 2 as cases with less than 50% keratinization, and Grade 3 as cases devoid of keratinization, i.e., non-keratinizing. Unstained, 3-µm-thick FFPE sections from the 100 surgical samples (50 SCC and 50 controls) were evaluated for HR-HPV by p16 IHC and DNA PCR. All p16-equivocal/positive and HPV DNA PCR-positive SCC were further evaluated for the expression of HPV-16 and HPV-18 mRNA transcripts using reverse transcription quantitative real-time polymerase chain reaction (RT-qPCR) to confirm transcriptionally active HPV.

#### Immunohistochemistry

Immunohistochemical expression of p16 was evaluated in all cases. In brief, deparaffinized FFPE sections of all cases were subjected to antigen retrieval using the Leica Bond protocol (Leica Biosystems) with proprietary Retrieval ER2 (ethylene diamine tetraacetic acid, pH 9.0) buffer for 20 min. A mouse monoclonal antibody against p16 (E6H4 clone, CINtec; Ventana Medical Systems, Arizona, USA) was utilized with a 1:4 dilution and detected with the Polymer Refine Kit (Leica Biosystems, New Castle, UK) on a Leica Bond autostainer. For positive immunohistochemical controls, a tonsil SCC with positive p16 expression was used. The threshold for p16 positivity was met in cases where ≥ 70% of tumor cells demonstrated strong diffuse nuclear and cytoplasmic staining. The threshold of ≥ 70% was used as it correlates with a positive HPV status in OPSCC [20]. Cases with moderate staining intensity in ≥ 70% of the cells or strong staining reactivity in ≥ 50 < 70% of the cells were categorized as *equivocal*.

#### Total DNA Extraction and Quality Assessment

Two to four 10-µm unstained sections from the FFPE blocks were collected in nuclease-free microcentrifuge tubes. Established precautions were followed to mitigate the contamination of the sections obtained for total DNA extraction. Following deparaffinization and proteinase K digestion overnight at 56 °C, total DNA was extracted using the Qiagen FFPE extraction kit (Qiagen, Hilden, Germany) according to the manufacturer’s instructions. The quality of the DNA extracted was assessed with a Biodrop µLite spectrophotometer (Biodrop LTD, Cambridge, UK). The absorbance ratios were used as quality indicators as follows: A260nm/A280nm (ca. 1.8 for pure DNA) and A260nm/A230nm (ca. 2.0–2.2 for pure DNA). The DNA was quantified using the Qubit dsDNA Broad Range Assay or Qubit dsDNA High Sensitivity kit and a Qubit 3.0 fluorometer (Life Technologies, Thermo Fisher Scientific, California, USA) according to the manufacturer’s recommendations. Lastly, the DNA integrity and functionality were determined by the amplification of a 205 base-pair human β-globin gene fragment, using conventional PCR and gel electrophoresis.

#### HR-HPV Detection and Genotyping

HPV DNA detection was performed using the HybriSpot HPV Direct Flow Chip kit (Master Diagnostica, Granada, Spain) according to the manufacturer’s recommendations. A total of 200-ng DNA were utilized for each reaction. The HybriSpot HPV Direct Flow Chip detects the following high-risk HPV genotypes: 16, 18, 26, 31, 33, 35, 39, 45, 51, 52, 53, 56, 58, 59, 66, 68, 73, and 82.

#### Total RNA Extraction and Quality Assessment

HR-HPV DNA-positive cases were selected for E6/E7 mRNA RT-qPCR. Total RNA was extracted from samples using the Maxwell® RSC RNA FFPE kit (Promega, Wisconsin, USA) and Maxwell RSC instrument (Promega, Wisconsin, USA), following the manufacturer’s instructions. RNA quality was assessed using a Biodrop µLite spectrophotometer (Biodrop LTD, Cambridge, UK). The quality indicators used were as follows: A260nm/A280nm (ca. 2.0 for pure RNA) and A260nm/A230nm (ca. 2.0–2.2 for pure RNA). RNA was quantified using the Qubit™ RNA High Sensitivity kit and a Qubit™ 4.0 fluorometer (Life Technologies, Thermo Fisher Scientific, California, USA) according to the manufacturer’s recommendations. For cDNA integrity and functionality quality control, GAPDH Taqman assay (Catalogue number Hs02786624_g1) and TaqMan Fast Advance Master Mix (Catalogue number 4444557) (Thermo Fisher Scientific, California, USA) were used according to the manufacturer’s recommendations. A Quant Studio 5 real-time PCR machine (Thermo Fisher Scientific, California, USA) was used for RT-qPCR.

Expression analysis of HPV-16 and HPV-18 mRNA transcripts with RT-qPCRA total of 100 ng of RNA was converted into cDNA using the High-Capacity cDNA Reverse Transcription kit (Thermo Fisher Scientific, California, USA), according to the manufacturer’s instructions. Quality control was maintained by including a “no reverse transcriptase” reaction for each RNA sample. HeLa cell mRNA confirmed HPV-16-positive samples served as positive controls for HPV-18 and HPV-16, respectively. HPV-16 and HPV-18 subtype-specific primers and probes were designed according to Lamarcq et al., 2002. Each qPCR contained 2-µl cDNA, 1X TaqMan Fast Advance Master Mix (Catalogue number 4444557) (Thermo Fisher Scientific, California, USA), 0.25-µM forward primer, 0.25-µM reverse primer, and 0.125-µM probe and nuclease-free water. qPCR were done in triplicate on a Quant Studio 5 real-time PCR machine (Thermo Fisher Scientific, California, USA) using the following cycling conditions: 2 min at 50 °C, hold at 95 °C for 2 min, 1 s at 95 °C for 40 cycles, and 20 s at 60 °C [21].

### Data Analysis

The statistical analysis was performed using SPSS Statistics version 27 (IBM, New York, USA). The associations between HR-HPV and SCC of the lip were assessed using Fisher’s exact two-sided tests. Demographic variables were also tested for association in the SCC and control group using T tests for continuous variables and the Fischer exact test for categorical variables.

## Results

### Demographics and Histology

The studied cohort of lip SCC consisted of 50 cases with 38 men and 12 women with a mean age of 61 years (range: 32 years to 95 years). The male-to-female ratio was 3:1. Tumors were mostly found on the vermillion border of the lip (*n* = 33), while 10 cases had tumor on the skin and in 7 cases the exact location of the tumor could not be determined. Of the 23 cases with documented HIV status, three participants were HIV positive. 28 SCC were classified as G1 (well differentiated), 18 as G2 (moderately differentiated), and 4 as G3 (poorly differentiated or non-keratinizing). There were no cases of verrucous carcinoma.

### p16 Immunohistochemistry

P16 immunohistochemistry was positive in 22% (*n* = 11) and equivocal in 4% (*n* = 2) of the SCC (Table 2). p16 IHC was negative in all cases in the control group. Of 11 p16-positive cases, 64% (*n* = 7) were located on the vermillion border of the lip, 18% (*n* = 2) were located on the skin and in 18% (*n* = 2), the site could not be determined. There was no significant association of p16 IHC positivity with age, gender, HIV status, site of biopsy, or histology grade (*p* > 0.05).

**Table 2:** p16-positive/equivocal SCC

Age	Gender	p16 expression	Lesion site on lip	Grade of tumor	HPV DNA PCR
56	F	50–70%	Vermillion	G1	HPV-18
44	M	> 70%	Vermillion	G1	Negative
80	F	> 70%	Indeterminate	G3	HPV-16
45	M	> 70%	Skin	G3	Negative
55	F	> 70%	Vermillion	G2	Negative
63	M	> 70%	Vermillion	G1	Negative
86	M	> 70%	Vermillion	G1	Negative
32	M	> 70%	Vermillion	G1	Negative
83	M	> 70%	Indeterminate	G3	Negative
51	M	> 70%	Vermillion	G1	Negative
63	M	> 70%	Vermillion	G1	Negative
50	M	> 70%	Skin	G1	Negative
46	M	50–70%	Indeterminate	G2	HPV-16

### HR-HPV DNA PCR Results

In the SCC group, HR-HPV DNA was identified in 10% of cases (*n* = 5). Among these, HPV-16 was the most prevalent type, detected in 3 SCC with single cases of HPV-18 and 33. In the control group, HR-HPV was present in 8% of cases (*n* = 4), with HPV-16 identified in 3 and HPV-45 in 1 case. Notably, no statistically significant association was found between HR-HPV PCR positivity and SCC of the lip (*p* = 1.000). Furthermore, there were no significant associations observed between HR-HPV DNA positivity and age (*p* = 0.631), gender (*p* = 0.876), location of tumor (*p* = 0.113), and HIV status (*p* = 0.530).

### Dual HR-HPV DNA and p16 IHC Positivity

One lip SCC (2%) with indeterminate biopsy site showed positivity for both HR-HPV DNA and p16 (Table 2). Two SCC with equivocal p16 staining were HR-HPV DNA positive, HPV-18 was found in one case, and HPV-16 was found in the other. The p16-positive/HR-HPV DNA-positive SCC was non-keratinizing (grade 3) and occurred in an 80-year-old female.

### HR-HPV mRNA Results

**Fig. 1:**
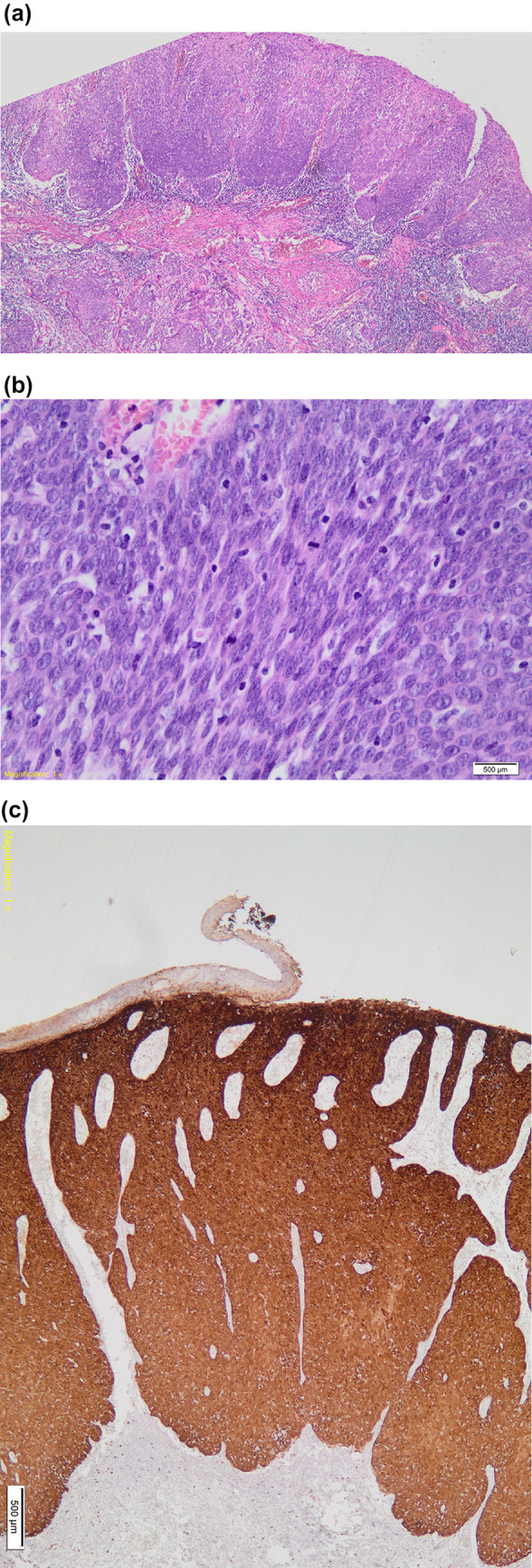
(**A**; H&E 40x) Infiltrating SCC with non-keratinizing histomorphology and dense lymphoid stroma associated with a basaloid in situ component. (**B**; H&E 40x) An island of atypical basophilic tumor cells. (**C**; 40x) p16 immunohistochemistry showing strong cytoplasmic and nuclear positive staining in > 70% of cells

HR-HPV mRNA PCR analysis was performed on the three p16-equivocal/positive and HPV DNA PCR-positive SCC (Table 3). HPV-16 mRNA was identified in the p16-positive/HPV-16 DNA-positive SCC. The latter case displayed a non-keratinizing morphology (categorized as G3) (Fig. 1A, B, C). Of the 11 p16-positive lip SCC, only one case demonstrated mRNA positivity, resulting in a low positive predictive value of 9% for p16 (sensitivity = 100 and specificity = 83%).

**Table 3:** mRNA results for HR-HPV DNA-positive and p16-equivocal/positive SCC

Age	Gender	p16 expression	HPV DNA PCR	Site on lip	Grade	mRNA	Smoker	HIV
56	F	50–70%	HPV-18	Vermillion	G1	Negative	Positive	NK
80	F	> 70%	HPV-16	Undetermined	G3	Positive (HPV-16)	NK	NK
46	M	50–70%	HPV-16	Undetermined	G2	Negative	NK	Negative

*NK* not known

## Discussion

SCC of the lip is a relatively common epithelial malignancy in sub-Saharan Africa with significant morbidity and mortality [22]. While known risk factors such as tobacco use, alcohol, ultraviolet light, and fair skin have been identified, there still exists a gap in our understanding of the disease’s etiopathogenesis [3, 4]. The role of HR-HPV in SCC of the lip has been a subject of investigation. A few studies (Table 1) have researched the role of HR-HPV in SCC of the lip and many with small sample sizes (some with a combination of SCC from the lip and other parts of the oral cavity) and utilizing single testing methodologies, predominantly HPV DNA PCR [12, 15–18]. The purposes of the present study were (1) to determine the prevalence of HR-HPV in a series of lip SCC from South African patients, using currently accepted HPV-testing methodologies (p16 IHC, PCR-based HPV-typing and mRNA PCR in a subset) and (2) to define the clinical and histomorphologic features of HPV-associated lip SCC, which may provide new insights into the development of these lesions.

To the best of our knowledge, our study is the first to use a multimodal testing approach, and the first to examine the incidence of HR-HPV in lip SCC using mRNA PCR technology. Using this approach, we were able to establish the presence of HR-HPV in 2% of the lip SCC.

In general, DNA PCR-based studies have reported a higher prevalence of HR-HPV in SCC of the lip. Mitsuishi et al. reported a prevalence rate of 100% using DNA PCR to detect HR-HPV in a small sample of 5 lip verrucous carcinomas, 3 of which harbored HPV-18 [17]. However, PCR detection alone does not distinguish HPV infections that are transcriptionally active from those that are not (i.e., so-called “passenger” HPV). Of the five HR-HPV DNA-positive SCC in the present study, only one was positive for p16 and mRNA PCR. In addition, HR-HPV DNA was detected in 8% of the control cases, which is just below the frequency of 10% observed in lip SCC. The latter finding clearly suggests that HR-HPV DNA detection alone is of limited value to confirm HPV-positive lip SCC.

p16 immunohistochemistry is easily accessible and affordable [23] and is recommended by the College of American Pathologists (CAP) as a reliable test for HR-HPV in OPSCC [20]. Transcriptionally active HR-HPV infection in OPSCC is typically correlated with p16 protein overexpression [20]. In an Egyptian study, Tealab et al. using p16 IHC demonstrated a high prevalence of HR-HPV in lip SCC (48%) [16].

Although the sensitivity of p16 IHC for HR-HPV infection is high, approaching 100%, its specificity is only 80–90%, as mechanisms other than pRb inactivation (e.g., pRb mutation) may lead to p16 overexpression [24]. In addition, while the concordance rate between p16 overexpression and molecularly confirmed HR-HPV infection is approximately 92–100% in OPSCC in the western world [25, 26], the concordance rates are significantly lower (32%), in regions with lower prevalence of HR-HPV-associated OPSCC, such as South Africa [27]. Therefore, p16 is not a perfect stand-alone surrogate marker for HR-HPV infection, especially in sub-Saharan Africa, where a low prevalence rate of HPV-associated OPSCC has been documented [27]. Similarly, of the 11 p16-positive lip SCC in our study (22%), only one case demonstrated mRNA positivity, resulting in a low p16 positive predictive value of 9% (sensitivity = 100 and specificity = 83%). Our results show that the combination of DNA PCR and p16 IHC appears to correlate with the presence of transcriptionally active virus (Table 3). A large meta-analysis showed a high sensitivity and specificity when both p16 IHC and HPV DNA PCR were utilized to detect transcriptionally active HPV infection in OPSCC [14].

Although HPV E6/E7 mRNA detection is the gold standard for identifying HR-HPV infection [14], the test is not widely available in developing countries due to technical and financial constraints. Similarly, to minimize study costs and based on p16’s outstanding negative predictive value (with p16 sensitivity approaching 100%) [24], we opted to evaluate the expression of HPV-16 and HPV-18 mRNA transcripts only in p16-equivocal/positive and HPV DNA PCR-positive lip SCC. Thus, the two p16-negative/HPV DNA PCR-positive lip SCC most likely represent passenger- and not truly active infections.

In fact, for pathology laboratories across sub-Saharan Africa, we recommend initial screening of lip SCC with p16 IHC complemented by HPV DNA PCR in p16-positive cases. Nevertheless, as clearly demonstrated in our study, mRNA ISH/PCR tests are best reserved for lip SCC with equivocal p16 IHC (Table 3).

Several factors may contribute to the lower HR-HPV prevalence in our study. This includes an increase in the prevalence of other risk factors in the population, such as tobacco and alcohol use, the African sun or regional sexual practices with a low prevalence of oral-genital sexual intercourse, leading to decreased exposure to HR-HPV in the oral cavity [28]. Additionally, genetic protective factors may play a role, as observed in cervical cancer [28].

According to the Joint United Nations Program on HIV/AIDS (UNAIDS), South Africa has one of the highest HIV prevalence rates, with a prevalence of 20.4% [29]. HIV infection has been linked to an increase in head and neck cancers. Beachler et al. reported an increase in HNSCC in HPV (oropharyngeal) and non-HPV (non-oropharyngeal locations, including the lip)-related sites in HIV-positive individuals compared to the general population [30]. Similarly, studies conducted in America and Netherlands have demonstrated a higher prevalence of HPV infection in HIV-positive individuals than in HIV-negative individuals [31, 32].

Despite the high HIV prevalence in certain regions in Africa, studies have revealed a lower-than-expected prevalence of HR-HPV in head and neck cancers, implying the involvement of multiple factors in the development of head and neck cancer in this area [27, 33]. In fact, none of the three SCC from HIV-positive patients in the present study were HR-HPV positive.

Numerous studies have consistently demonstrated a strong association between HR-HPV and non-keratinizing histomorphology in OPSCC [8, 9]. Similar to HPV-associated OPSCC, the only HR-HPV-positive lip SCC in our study showed a non-keratinizing morphology.

Most of the p16-positive lip SCC in our study arose from the vermillion border of the lower lip, a transition from highly keratinized external skin to less keratinized internal skin. Given that the majority of HPV-associated squamous lesions of the uterine cervix occur at the ecto-endocervical transition zone, it is tempting to speculate that the vermillion border of the lower lip might bear some analogy to the latter. However, an exact site for the only HR-HPV-positive case in our study could not be determined.

The strength of our study lies in the fact that, it is the first prevalence study using multiple HPV-testing modalities, including mRNA PCR in lip SCC. However, a notable limitation is the retrospective nature of the study with limited information on participant risk factors.

## Conclusion

While the oropharynx remains the most common site in the head and neck where HR-HPV-associated SCC is known to occur, the findings from our cohort of lip SCC suggest that HR-HPV may have an insignificant role in the pathogenesis of SCC at this site (prevalence rate of 2%). However, the findings of the present study contribute to the growing body of evidence, supporting the presence of HR-HPV in SCC of the lip, underscoring the need for larger multi-institutional studies to better understand this relationship. Due to its low ppv (9%), p16 IHC is insufficient to establish HR-HPV infection in SCC of the lip. The combination of DNA PCR and p16 IHC appears to correlate with the presence of transcriptionally active virus. HPV E6/E7 mRNA detection is the gold standard for identifying HR-HPV infection. E6/E7 mRNA tests are not widely available in sub-Saharan Africa due to technical and financial constraints and maybe best reserved for lip SCC with equivocal p16 IHC.

## Data Availability

All datasets and research materials are available for revision on request.
